# Are Inductive Current Transformers Performance Really Affected by Actual Distorted Network Conditions? An Experimental Case Study

**DOI:** 10.3390/s20030927

**Published:** 2020-02-10

**Authors:** Alessandro Mingotti, Lorenzo Peretto, Lorenzo Bartolomei, Diego Cavaliere, Roberto Tinarelli

**Affiliations:** Department of Electrical, Electronic and Information Engineering–Guglielmo Marconi, Alma Mater Studiorum, University of Bologna, 40136 Bologna, Italy; lorenzo.peretto@unibo.it (L.P.); lorenzo.bartolomei@unibo.it (L.B.); diego.cavaliere2@unibo.it (D.C.); roberto.tinarelli3@unibo.it (R.T.)

**Keywords:** actual waveforms, inductive current transformers, measurement setup, composite error, accuracy, uncertainty, electrical measurements, total harmonic distortion

## Abstract

The aim of this work is to assess whether actual distorted conditions of the network are really affecting the accuracy of inductive current transformers. The study started from the need to evaluate the accuracy performance of inductive current transformers in off-nominal conditions, and to improve the related standards. In fact, standards do not provide a uniform set of distorted waveforms to be applied on inductive or low-power instrument transformers. Moreover, there is no agreement yet, among the experts, about how to evaluate the uncertainty of the instrument transformer when the operating conditions are different from the rated ones. To this purpose, the authors collected currents from the power network and injected them into two off-the-shelf current transformers. Then, their accuracy performances have been evaluated by means of the well-known composite error index and an approximated version of it. The obtained results show that under realistic non-rated conditions of the network, the tested transformers show a very good behavior considering their nonlinear nature, arising the question in the title. A secondary result is that the use of the composite error should be more and more supported by the standards, considering its effectiveness in the accuracy evaluation of instrument transformers for measuring purposes.

## 1. Introduction

In recent years the operation of Instrument Transformers (ITs), either the legacy inductive type and the new Low-Power Instrument Transformers (LPITs), has been affected by a huge revolution in the power network. In particular, the spread of Renewable Energy Sources (RESs) among all voltage levels; the massive installation of all kinds of energy meters; and last, but just from a chronological point of view, the increasing presence of electric vehicles in the low-voltage (LV) level are affecting the power quality of the network. Poor power quality leads to severe and unwanted consequences, both on the electrical and electronic assets, as well as on end-users. Distorted voltages and currents can cause luminous flux variations in lamps and serious consequences on human beings [1,2,3]. Furthermore, bad power quality affects the reliability of electrical and electronic components, leading to heating effects or degradation phenomena of insulation materials, or the combination of both [4,5,6]. In order to correctly and properly address such issues, the measuring instruments used for evaluating Power Quality need to operate accurately in a large frequency bandwidth. In particular, representing the weak elements in the measurements chain for such purposes, they shall provide accurate measurements in all the previous mentioned conditions. That is why a Standard dedicated to the use of ITs for power quality measurements has been developed: the IEC 61869-103 [7].

Moreover, users and manufacturers should be guided on how to test ITs to verify their correct operation and level of uncertainty, also, in such off-nominal operating conditions (a starting point for the tests is provided in ref. [7]). Focusing on ITs, in literature several other works, the Standards, dealing with them, can be found. Starting from the Standards, the main series is the IEC 61869 where 61869-1 [8] deals with general requirements on ITs, while 61869-2, -3 and -4 are specifically written for current, voltage and combined transformers (CTs, VTs and CVTs), respectively [9,10,11]. The same structure has been adopted also for LPITs, but they are out of the aim of this work.

The ITs’ modeling can be considered quite developed to date; in fact, several works are present in literature and new material is always being published [12,13,14,15,16,17,18]. However, the approaching of ITs from their modeling does not always provide significant results in all fields of interest, such as in the case of ITs working at off-nominal conditions. The studies on this particular aspect are spreading in recent years, due to their relevance in the overall performance of ITs. For example, in [19,20,21,22] the effect of temperature on IT has been studied, while construction methods and conducted disturbances are analyzed in [23,24,25].

With the present work, instead, the authors want to obtain a bifold result. In fact, the aim is to understand whether the CTs are really affected, in terms of accuracy, by operating conditions different from the rated ones, but realistic. Furthermore, in doing so, several distorted test waveforms, having different levels of total harmonic distortion (THD), have been collected from the grid to be used in this work as realistic test waveforms. The underlying idea that supports this choice comes from the need of having a common set of distorted test waveforms to be applied to the ITs. This is something that is not provided in the available standards; hence they should move in such a direction. However, the literature tackling the CTs working under off-nominal power conditions is really vivid. For example, some testing procedures are described in [26,27], while error and non-linearity correction have been studied and proposed in [28,29,30,31]. Finally, accuracy measurements and calibration procedures are discussed in detail in [32,33,34,35].

In light of the above, this work uses the literature as a starting point to raise and to study the issue of which of the effects are really causing the power network off-nominal conditions on CTs in realistic experimental conditions.

Authors started in [36] by using COMTRADE files [37] with distorted transient waveforms collected in the field (from faulty operating conditions). Such waveforms were injected into a CT to assess its performance.

In this paper, instead, steady-state actual distorted waveforms have been collected from the grid and then applied to two CTs typically implemented in the Medium Voltage (MV) network. Afterwards, the performance of the CTs have been evaluated through the use of the well-known composite error and of an approximated version of it. This choice has been supported by the fact that the well-known frequency response analysis is not particularly efficient to assess the accuracy performance of a nonlinear instrument like the inductive CT.

Such a way to assess the CT’s accuracy is typical of, but not limited to, protective ITs. For example, a virtual instrument has been developed in [38]; in [39] a model has been studied to assess ITs affected by power quality issues; [40,41] instead, describe the use of ratio error applied to each harmonic component and the application of the frequency response approach, respectively. The application of the composite error on the evaluation of the ITs’ performance in a variety of network conditions is studied in [35,42,43]; finally, the relevance and criticality of the accuracy when dealing with ITs is confirmed by [44,45,46,47,48,49].

The paper is structured as follows: Section 2 contains the complete description on how the actual steady-state distorted signals have been acquired. Section 3 describes the simple measurement setup implemented to test the inductive CTs. The main tests performed are listed in Section 4, while in Section 5 the results and the postprocessing analysis are included. Finally, a brief conclusion with the key points is drawn in Section 6.

## 2. Acquisition of Actual Signals

**Figure 1:**
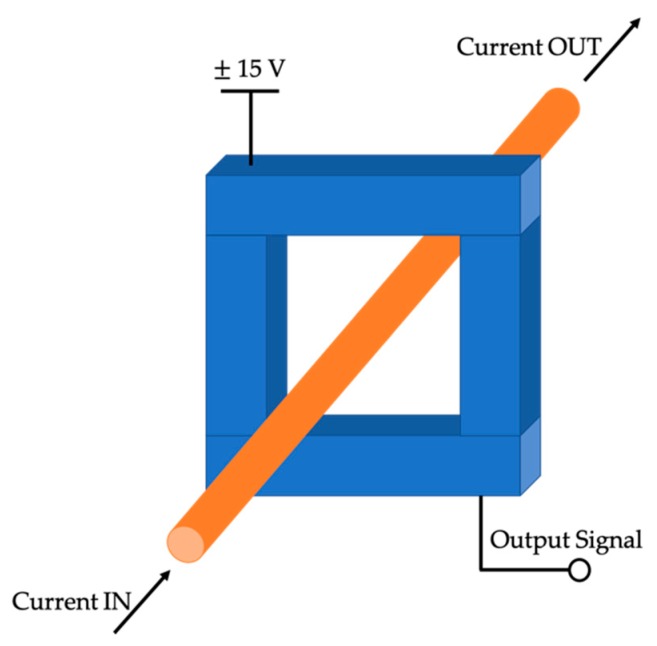
Measurement instrument developed for the acquisition of actual currents.

Two off-the-shelf CTs have been tested using actual signals. Before describing the CT testing (see Section 4), it is necessary to describe how such signals have been acquired. First of all, the measurement instrument developed for the current collection is depicted in Figure 1.

It simply consists of a Hall-effect-based current sensor, a direct current (DC) generator to supply it, and a data acquisition board (DAQ) to collect the samples. The current sensor is a LEM LA 100-P with a primary measuring range of 0–150 A; a secondary nominal current of 50 mA and an accuracy of ±0.45%. As for the DAQ, an NI 9238 has been used. Its main characteristics are summarized in Table 1.

Since the DAQ supports only voltage inputs, and the LEM LA 100-P provides a current output, a resistor has been inserted as current sensor’s load. Then, the voltage across the load has been acquired by means of the DAQ. The setup has been then used, inside the laboratory environment, to acquire currents flowing along the LV network when using different instrumentation; e.g., a thermostatic chamber, air conditioner, calibrator, power source, etc. According to the current sensor’s specifications, the measurand expected magnitude and the DAQ’s max input signal, a 100 Ω load resistance has been chosen. The acquired signals have been sorted in terms of THD, because the aim is to have signals with a variety of actual harmonic content, regardless the source of that content. Hence, values in Table 2 are listed from signals A to E for the sake of simplicity. The collected signals have all a THD < 10%, which is a plausible and realistic distortion for the currents absorbed by users in LV and MV systems, according to IEEE Std. 519-2014 [50].

**Figure 2:**
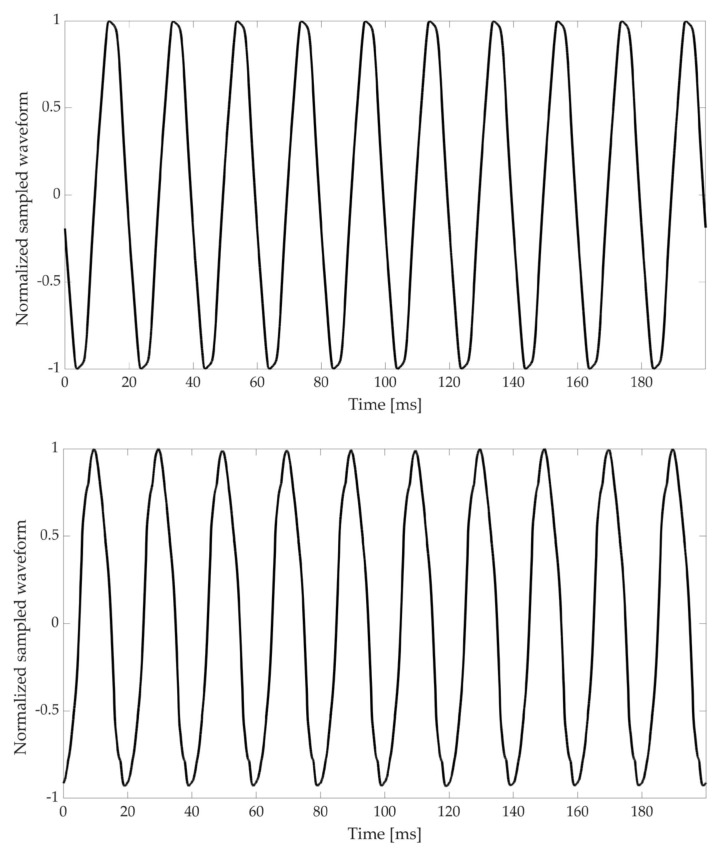
Waveform of the signal A (**top**) and E (**bottom**). The two sampled waveforms are normalized to 1.

As an example, the waveform of signals A and E have been plotted and presented in Figure 2. It is worth the effort to highlight that, within the limits fixed in [50], it is not straight forward to recognize the level of distortion of a signal. The same comment can be extended to the voltages of the network, the THD limits for which MV and LV networks are defined in the EN (European standards) 50,160 [51], and are even more strict compared to those in [50].

## 3. Measurement Setup for CT Testing

To inject the current, acquired as described in Section 2, the following measurement setup has been improved and adopted. It consists of:

- A 14-bit Keysight Function/Arbitrary waveform generator 33,220A. It features a frequency resolution of 1 μHz, a frequency accuracy of ±(20 ppm + 3 pHz), and a sampling frequency of 50 MSa/s. The function generator has been used to replicate the current waveform previously collected.
- Fluke Transconductance 52,120A. Its task is to transduce the output voltage of the generator into a current consistent with the rated values of the transformers under test (TUTs). The main accuracy parameters of the transconductance are listed in Table 3.
- The MV TUTs. Their main characteristics are collected in Table 4.
- Two shunt resistors to measure both the primary and secondary currents of the TUTs. The first, S1, is a 1 mΩ resistor, and it is installed in series to the primary current; the second resistor, S2, has a 10 mΩ resistance, and it is installed in series to the secondary current and to a 220 mΩ/7 W resistive burden to guarantee the TUT’s operation under rated conditions. As for their uncertainty, they feature 0.01% and 0.005% for S1 and S2, respectively. No other information is available from the manufacturer. Nevertheless, the characterization described in the next Section has provided the information necessary for the TUTs’ proper testing. Finally, in [36] the two shunts have been characterized vs frequency to assess whether they are affected from it or not. The results showed that the frequency does not affect both shunts, reporting variations lower than $2\times 10^{-6}$ Ω.
- An NI9238 DAQ is used to acquire the output voltages of both the shunts.

**Figure 3:**
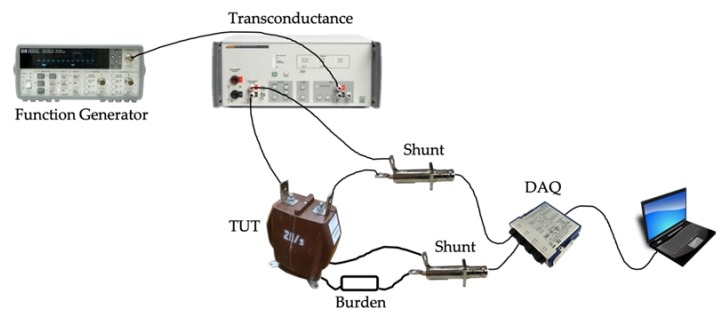
Schematic of the measurement setup used for the tests.

The conceptual scheme of the measurement setup is depicted in Figure 3.

To summarize the operation of the setup, the signals listed in Table 2 have been reproduced with the function generator, transduced by the transconductance amplifier 52,120A and then injected into the TUT.

## 4. Description of the Tests

The aim of the test is to acquire both primary and secondary currents of the TUTs. The currents are then used to perform an accuracy evaluation of the TUTs. First, the signals in Table 2 have been continuously fed from the function generator to the transconductance. Second, the output of the transconductance has been set to values of currents in accordance with [9]. In particular, [9] states that the ratio and phase errors, used to evaluate the accuracy class of the transformers, are evaluated at 5%, 20%, 100% and 120% of the transformer’s rated primary current.

Consequently, for the adopted TUTs, a set of RMS currents of 20, 4 and 1 A for T1 and of 100, 20 and 5 A for T2 have been generated by the transconductance and injected into the TUT. The 120% current test has been omitted due to the limitations of the transconductance; however, over currents are not the aim of this work, which tackles the normal operation of the network in actual conditions.

To measure the currents, two shunts have been adopted together with a DAQ NI9238 in order to collect the output voltages. The whole measurement chain composed by the shunts and the DAQ has been characterized before and after the tests to ensure its repeatability and to confirm the shunt resistance values. The characterization process has been done by injecting the current of interest I (5%, 20% and 100% of the primary and secondary currents) into the shunts by using a reference calibrator (Fluke 6105A), and then by reading the voltage measurements from the DAQ. One hundred measurements for each current level have been acquired, and the mean value $R_{m}$ of the “shunts + DAQ” chain conversion factor has been computed, whose measurement unit is the ohm. The results of the characterization provided the estimate values of the equivalent resistances of the “shunts + DAQ” chain for each measured current. Such resistance values, used to compute the currents from the measured voltages, and their related extended uncertainty $u_{R}$ (coverage factor K=2), are listed in Table 5. The table includes the values of the first characterization, considering that the second characterization provided the same results.

As for $u_{R}$, it has been computed by means of the propagation of the uncertainties, as described in the Guide to the expression of Uncertainty in Measurement [52]:

(1) $$u_{R}=\sqrt{{\left(\frac{\partial R}{\partial V}\right)}^{2}u_{\mathrm{Va}}^{2}+{\left(\frac{\partial R}{\partial I}\right)}^{2}u_{\mathrm{Ib}}^{2}}=\sqrt{\left(\frac{u_{\mathrm{Va}}^{2}}{I^{2}}\right)+{\left(-\frac{V_{m}}{I^{2}}\right)}^{2}u_{\mathrm{Ib}}^{2}}\;$$

where:

- $V_{m}$ is the average measured voltage across the shunt;
- $u_{\mathrm{Va}}$ is the uncertainty of the measured voltage evaluated with type A method, as the standard deviation of $V_{m}$;
- $u_{\mathrm{Ib}}$ is the uncertainty of the generated current evaluated with the type B method starting from the accuracy specification of the calibrator (with transconductance for the case of 100 A).

For the sake of completeness, the type A and type B methods are described in [52] as methods to evaluate uncertainty due to random or systematic effects, respectively. The former method is based on the estimation of the expected value of the measurand and of its standard deviation, starting from *N* measurements. In the latter method instead, the contributions to the uncertainty are provided by the manufacturer of the devices in terms of indices. These are basically two: the error on the full scale and the one on the reading.

There are few considerations regarding (1) to be pointed out: first, the measured voltage $V_{m}$ and the generated current I are obviously uncorrelated quantities; second, the type B evaluated uncertainty $u_{Vb}$ does not appear in (1), since the whole “shunt + DAQ” measurement chain is the system to be characterized.

In other words, the value of $R_{m}$ already embodies the contribution due to the DAQ’s error in every considered current scenario. This operation was possible because of the fact that exactly the same setup has been implemented both in the characterization and in the measurement procedures.

Afterwards, for each signal of Table 2, 100 measurements of 10 periods of the above-mentioned set of currents have been collected for both the primary and the secondary by acquiring the voltage drop on the shunts S1 and S2 (sampling frequency 50 kSa/s).

To complete the set of tests, and to better evaluate the results, the two TUTs have been fed with sinusoidal signals. In particular, the three currents of interest (100, 20, 5 A and 20, 4, 1 A) have been injected as 50 Hz sinusoidal waveforms. Again, 100 measurements of both primary and secondary currents have been collected. This last test has been considered fundamental to assess the performance of the CTs at rated conditions; hence, to use the results as a comparison with the other operating conditions.

## 5. Experimental Results

The evaluation of the TUTs accuracy has been carried out by means of the ratio error ε, the phase error Δφ, and the composite error $\varepsilon_{c}$, defined in [9]. The indices ε and Δφ are used to assess the performance of the CTs at sinusoidal conditions, whilst $\varepsilon_{c}$ is introduced after the encouraging results obtained in [36]. In fact, $\varepsilon_{c}$ was used in [36] to assess the CT’s behavior in the presence of fault-derived signals; while in this work, $\varepsilon_{c}$ is applied to the evaluate CTs in presence of steady-state distorted signals. For convenience, the formula of $\varepsilon_{c}$ is reported:

(2) $$\varepsilon_{c}≜\;\frac{\sqrt{\frac{1}{T}\int_{0}^{T}{\left(k_{r}i_{s}{-i}_{p}\right)}^{2}dt}}{I_{p}}\cdot 100\%\;$$

where:

- $k_{r}$ is the rated transformation ratio;
- $i_{p}$ is the instantaneous value of the primary current;
- $i_{s}$ is the instantaneous value of the secondary current;
- $I_{p}$ is the RMS of the primary current;
- T is the duration of one cycle.

If $i_{p}$ and $i_{s}$ are sinusoidal waveforms, then the approximated composite error $\varepsilon_{c}^{*}$ can be calculated with (see [43]):

(3) $$\varepsilon_{c}^{*}\approx \sqrt{\varepsilon^{2}{+\;\Delta \varphi }^{2}}\;.$$

Attention shall be paid to the usage of ε and Δφ: these two parameters are defined for the instrument transformers only in the presence of sinusoidal quantities. When actual distorted waveforms are considered, as done below, ε and Δφ are computed for the 50 Hz components. This is a nonconventional procedure, according to the ε and Δφ definitions, even if commonly adopted.

In Table 6 and Table 7 the results are reported for T1 and T2, respectively. The following quantities are shown: ε and Δφ for the 50 Hz harmonic component; $\varepsilon_{c}$ computed by the numerical implementation of (2) and $\varepsilon_{c}^{*}$. Each quantity is averaged over the 100 repeated measurements conducted for each signal of Table 2 at 5%, 20% and 100% of the rated primary current $I_{\mathrm{pr}}$.

**Figure 4:**
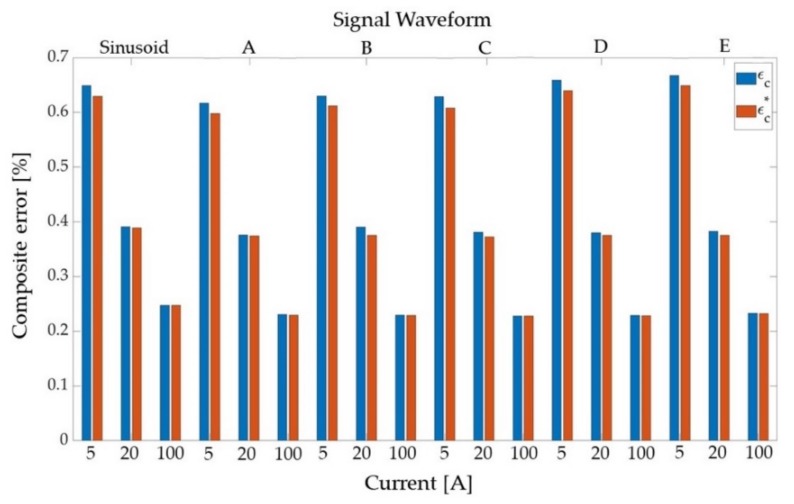
Graphical representation of the results in Table 7.

From the graphical representation of Table 7 in Figure 4, it emerges that $\varepsilon_{c}^{*}$ slightly underestimates $\varepsilon_{c}$ for T2. It is also evident that the composite error decreases as the current gets closer to the rated one, which is an expected behavior, and that the composite error variation among the different distortion cases is almost absent. As for T1, the same observations can be drawn from Table 6, and consequently, their graphical representation is omitted.

For convenience, the maximum standard deviations of the averages of ε, Δφ, $\varepsilon_{c}$ and $\varepsilon_{c}^{*}$, for every current case, is reported in Table 8. The standard deviation of each parameter for both the TUTs gets smaller as the primary current gets closer to the rated one. The composite error and the ratio error are defined as dimensionless percentage quantities; thus, their standard deviation shall be expressed accordingly as percentages.

The uncertainty of the parameters in the Table, evaluated with the type B method (as described in [52]), has been omitted, because, for the comparison of the signals from A to E, in each current category, the measurement chain involved is the same. Therefore, for the evaluation of the parameters’ variation, the uncertainty evaluated with type B method is not significative.

In Table 6 and Table 7, note that both T1 and T2 are compliant with their rated accuracy class (0.5 and 0.2, respectively): the measured ratio and phase errors in the sinusoidal case are smaller than the limits prescribed in [9]. The limits for the accuracy classes 0.2 and 0.5 are reported in Table 9, showing the maximum admitted ratio error $\varepsilon_{max}$ and phase error ${\Delta \varphi }_{max}$. Since these limits are defined for sinusoidal waveforms, then it is possible to extend Table 9 by applying (3) in order to estimate the corresponding composite error limits $\varepsilon_{c_{max}}^{*}$ for each accuracy class. These values have been computed and listed in the last three columns of Table 9.

At this point, it is interesting to compare the numerically computed values of $\varepsilon_{c}$ with the corresponding value of $\varepsilon_{c_{max}}^{*}$, since it could be a criterion to assess the TUTs accuracy performance in the steady-state distorted conditions. The composite error physical meaning is how well the instrument transformer output matches the measurand, and $\varepsilon_{c_{max}}^{*}$ is the estimate of the worst tolerated scenario in sinusoidal conditions. Therefore, if $\varepsilon_{c}$ in the distorted cases is smaller than $\varepsilon_{c_{max}}^{*}$, than the TUT accuracy could be considered acceptable.

The results in Table 6 and Table 7 shows that both TUTs present a composite error $\varepsilon_{c}$ smaller than the limit value $\varepsilon_{c_{max}}^{*}$ in all of the distorted cases. Additional tests were carried out to support the results herein obtained: the first, at very high THD (25.0%) with random frequency components up to the 20th harmonic order; the second, at THD = 10%, but with a single frequency component which was changed from the 20th (1000 Hz) to the 100th (5000 Hz) harmonic order. Under these conditions the TUTs have maintained their accuracy class, showing consistency with the results obtained in Table 6 and Table 7. As a further comment, the instrument current transformer performance evaluated by means of the composite error suggests that its accuracy is mainly dependent upon the performance at 50 Hz and only (very) slightly affected by the actual harmonic content of the measurand.

It is worth highlighting that all the above-mentioned results prove the applicability of (3) for two main reasons. First, the set of currents used has an actual harmonic content consistent with the Standards. Second, the peculiarity of using only the 50 Hz component is supported by the fact that common measurement instruments (e.g., Phasor Measurement Unit, Energy Meters, etc.) already extract such a component.

To validate the obtained results, the measurements have been repeated after more than a month to ensure also their repeatability. The new set of results completely confirms what was already presented in this section.

As a final and main comment, it is interesting to assess the obtained results from a practical point of view. Such results allow to state that CTs are not affected by realistic distortions/off-nominal conditions of the network up to a 25% of THD. Furthermore, the use of $\varepsilon_{c}^{*}$ and $\varepsilon_{c}$ for measuring IT is supported by the results and by the fact that, at off-nominal conditions, they provide a more significant information compared to the ratio and phase errors.

## 6. Conclusions

The aim of the work is to raise the issue of whether or not realistic power network conditions affect the accuracy performance of inductive current transformers. This has been done considering that no standard defines how to proceed with such tests, which waveforms have to be injected, and how much they are affected. Therefore, actual distorted currents have been collected from the grid and injected to two off-the-shelf current transformers.

Afterwards the results have been evaluated in terms of the well-known composite error and an approximated version of it. The former index is typically adopted for protective instrument transformers. From the results it emerges that both transformers show really good behavior at rated and at off-nominal conditions. Hence, in this particular case study, which involves devices adopted by several utilities, it is reasonable to ask whether the influence of distorted signals (with values within the limits suggested by IEEE std 519-2014) is really affecting the behavior of the transformers. The answer to that question, from the presented results, is no. In other words, in practical cases, hence, when the transformer is operating at actual conditions, their behavior is only slightly affected by the distorted input signals. Such a conclusion is not to be claimed as obvious, because the common idea is that inductive CTs operation is quite affected by distorted primary currents. As a consequence, several works in literature deal with how to solve such an issue despite is minor significance in realistic conditions (even if the proposed solutions are typically effective).

A secondary conclusion is that, in light of the previous one, the application of the composite error should be encouraged for the assessment of the transformers’ accuracy when they operate at off-nominal conditions. As a matter of fact, it provides a more exhaustive information on the accuracy of the transformer, compared to the one of ratio and phase errors.

Overall, on the one hand, standards should include more details for the users regarding how to test the instrument transformers in more realistic conditions. On the other hand, such realistic conditions should be tackled as much as they influence the accuracy of the transformers; and hence, not considered if it is not the case.

## Figures and Tables

**Table 1:** Main features of the data acquisition board (DAQ) NI 9238.

Architecture	24-bit	Max Input Signal	±500 mV
**Sample rate**	50 kS/s/ch	**Simultaneous channels**	YES
**ADC**	Delta Sigma	**Temperature range**	−40 to 70 °C
**Gain Error**	±0.07%	**Offset Error**	±0.005%

**Table 2:** List of the acquired signals and their total harmonic distortion (THD).

Signal	THD [%]
**A**	4.5
**B**	7.2
**C**	7.7
**D**	8.7
**E**	9.9

**Table 3:** Main features of the two transconductance 52120A.

Current Range	% of Output	% of Range
**2**	0.015	0.070
**20**	0.015	0.060
**120**	0.015	0.020

**Table 4:** Main features of the two transformers under test (TUTs).

TUT	Ratio [A]	Power [VA]	Accuracy Class	Extended Current Rating
**T1**	20/5	6	0.5	120% (24 A)
**T2**	100/5	6	0.2	120% (120 A)

**Table 5:** Results of the characterization of the “shunts + DAQ” measurement chain.

Shunt	I [A]	$R_{m}$ [mΩ]	$u_{R}$ [μΩ]
**S1**	1	0.999	1
	4	0.9995	0.4
	5	0.9994	0.3
	20	0.9994	0.1
	100	0.99941	0.07
**S2**	0.25	10.028	3
	1	10.029	1
	5	10.0291	0.6

**Table 6:** Results for T1. Averages of ε, Δφ, $\varepsilon_{c}$, $\varepsilon_{c}^{*}$ for the sinusoidal case and all the distorted signals, at 0.05 $I_{\mathrm{pr}}$, 0.2 $I_{\mathrm{pr}}$ and $I_{\mathrm{pr}}$.

$I_{p}\;[A]$	Signal	ε [%]	Δφ [mrad]	$\varepsilon_{c}\;[\%]$	$\varepsilon_{c}^{*}\;[\%]$
1	Sinusoid	−0.7865	19.53	2.148	2.105
	A	−0.8117	19.49	2.156	2.111
	B	−0.8172	19.81	2.181	2.143
	C	−0.7534	19.42	2.120	2.083
	D	−0.8070	19.47	2.150	2.107
	E	−0.8000	19.38	2.142	2.097
4	Sinusoid	−0.4752	12.497	1.3398	1.3370
	A	−0.4983	12.460	1.3448	1.3419
	B	−0.5216	12.568	1.3605	1.3607
	C	−0.4667	12.383	1.3239	1.3233
	D	−0.5195	12.324	1.3395	1.3374
	E	−0.4768	12.445	1.3345	1.3327
20	Sinusoid	−0.2778	7.580	0.8086	0.8073
	A	−0.2966	7.577	0.8181	0.8137
	B	−0.3090	7.598	0.8239	0.8202
	C	−0.2820	7.584	0.8123	0.8092
	D	−0.3027	7.636	0.8274	0.8214
	E	−0.2830	7.592	0.8146	0.8102

**Table 7:** Results for T2. Averages of ε, Δφ, $\varepsilon_{c}$, $\varepsilon_{c}^{*}$ for the 50 Hz case and all the distorted signals, at 0.05 $I_{\mathrm{pr}}$, 0.2 $I_{\mathrm{pr}}$ and $I_{\mathrm{pr}}$.

$I_{p}\;[A]$	Signal	ε [%]	Δφ [mrad]	$\varepsilon_{c}\;[\%]$	$\varepsilon_{c}^{*}\;[\%]$
5	Sinusoid	−0.176	6.04	0.649	0.629
	A	−0.173	5.73	0.617	0.598
	B	−0.192	5.81	0.630	0.612
	C	−0.190	5.77	0.629	0.608
	D	−0.234	5.96	0.659	0.640
	E	−0.219	6.11	0.667	0.649
20	Sinusoid	−0.0331	3.877	0.391	0.3891
	A	−0.0876	3.639	0.376	0.3743
	B	−0.0937	3.633	0.391	0.3752
	C	−0.0838	3.632	0.381	0.3727
	D	−0.0936	3.636	0.380	0.3754
	E	−0.1040	3.605	0.383	0.3752
100	Sinusoid	0.0541	2.4116	0.24734	0.24714
	A	−0.0206	2.2879	0.23080	0.22973
	B	0.0292	2.2702	0.22947	0.22889
	C	0.0378	2.2468	0.22811	0.22784
	D	−0.0130	2.2804	0.22875	0.22842
	E	−0.0330	2.2995	0.23260	0.23231

**Table 8:** $\sigma_{\varepsilon }$, $\sigma_{\Delta \varphi }$, $\sigma_{\varepsilon_{c}}$ and $\sigma_{\varepsilon_{c}^{*}}$ are the maximum standard deviations of the averages of ε, Δφ, $\varepsilon_{c}$ and $\varepsilon_{c}^{*}$, respectively, for both the TUTs at 0.05$\;I_{\mathrm{pr}}$, 0.2 $I_{\mathrm{pr}}$ and $I_{\mathrm{pr}}$.

TUT	*I* [A]	$\sigma_{\varepsilon }\;[\%]$	$\sigma_{\Delta \varphi }\;[\mathrm{mrad}]$	$\sigma_{\varepsilon_{c}}\;[\%]$	$\sigma_{\varepsilon_{c}^{*}}\;[\%]$
T1	1	0.0009	0.01	0.002	0.001
	4	0.0003	0.003	0.0005	0.0003
	20	0.0003	0.002	0.0003	0.0002
T2	5	0.002	0.01	0.002	0.001
	20	0.0004	0.004	0.003	0.0004
	100	0.0002	0.0006	0.00009	0.00007

**Table 9:** ε and Δφ limits for the 0.2 and 0.5 accuracy classes transformers defined in [2]. The table has been extended with $\varepsilon_{c_{max}}^{*}$ obtained from the limits $\varepsilon_{max}$ and ${\Delta \varphi }_{max}$.

Accuracy Class	$\varepsilon_{max}\;[\%]$			${\Delta \varphi }_{max}\;[\mathrm{mrad}]$			$\varepsilon_{c_{max}}^{*}\;[\%]$		
	0.05 $I_{\mathrm{pr}}$	0.20 $I_{\mathrm{pr}}$	$I_{\mathrm{pr}}$	0.05 $I_{\mathrm{pr}}$	0.20 $I_{\mathrm{pr}}$	$I_{\mathrm{pr}}$	0.05 $I_{\mathrm{pr}}$	0.20 $I_{\mathrm{pr}}$	$I_{\mathrm{pr}}$
0.2	0.75	0.35	0.2	9	4.5	3	1.17	0.57	0.36
0.5	1.5	0.75	0.5	27	13.5	9	3.09	1.54	1.03
